# Long-term progression of clinician-reported and gait performance outcomes in hereditary spastic paraplegias

**DOI:** 10.3389/fnins.2023.1226479

**Published:** 2023-09-22

**Authors:** Diana Maria Cubillos Arcila, Gustavo Dariva Machado, Valéria Feijó Martins, Vanessa Bielefeldt Leotti, Rebecca Schüle, Leonardo Alexandre Peyré-Tartaruga, Jonas Alex Morales Saute

**Affiliations:** ^1^Graduate Program in Medicine, Medical Sciences, Universidade Federal do Rio Grande do Sul, Porto Alegre, Brazil; ^2^Exercise Research Laboratory, Universidade Federal do Rio Grande do Sul, Porto Alegre, Brazil; ^3^Division of Medical Genetics, Hospital de Clínicas de Porto Alegre, Porto Alegre, Brazil; ^4^Graduate Program in Human Movement Sciences, Universidade Federal do Rio Grande do Sul, Porto Alegre, Brazil; ^5^Biostatistics Unit, Hospital de Clínicas de Porto Alegre, Porto Alegre, Brazil; ^6^Department of Statistics, Universidade Federal do Rio Grande do Sul, Porto Alegre, Brazil; ^7^Division of Neurodegenerative Diseases, Department of Neurology, Heidelberg University, Heidelberg, Germany; ^8^Division of Neurology, Hospital de Clínicas de Porto Alegre, Porto Alegre, Brazil; ^9^Neurogenetics, Clinical Research Center, Hospital de Clínicas de Porto Alegre, Porto Alegre, Brazil; ^10^Department of Internal Medicine, Faculdade de Medicina, Universidade Federal do Rio Grande do Sul, Porto Alegre, Brazil

**Keywords:** hereditary spastic paraplegias, gait analysis, clinical outcome assessment, performance outcomes, clinician-reported outcomes

## Abstract

**Introduction:**

Hereditary spastic paraplegias (HSPs) are a heterogeneous group of neurodegenerative diseases in which little is known about the most appropriate clinical outcome assessments (COAs) to capture disease progression. The objective of this study was to prospectively determine disease progression after 4.5 years of follow-up with different clinician-reported (ClinRO) and gait performance outcomes (PerFOs).

**Methods:**

Twenty-six HSP patients (15 SPG4, 5 SPG7, 4 SPG5, 2 SPG3A) participated in this single-center cohort study in which the ClinRO: Spastic Paraplegia Rating Scale; and the PerFOs: 10-meters walking test and timed-up and go (TUG), at self-selected and maximal walking speeds; Locomotor Rehabilitation Index; and 6-min walking test were performed at baseline and after 1.5 (18 patients) and 4.5 (13 patients) years.

**Results:**

In the 3-year interval between the second and third assessments, significant progressions were only found in PerFOs, while in the overall 4.5 years of follow-up, both PerFOs and ClinROs presented significant progressions. The progression slopes of COAs modeled according to the disease duration allowed the estimation of the annual progression of the outcomes and sample size estimations for future clinical trials of interventions with different effect sizes. TUG at maximal walking speed was the only COA capable of differentiating subjects with a worse compared to a stable/better impression of change and would require the smallest sample size if chosen as the primary endpoint of a clinical trial.

**Discussion:**

These findings indicate that both performance and clinician-reported outcomes can capture long-term progression of HSPs, with some PerFOs presenting greater sensitivity to change. The presented data are paramount for planning future disease-modifying and symptomatic therapy trials for this currently untreatable group of diseases.

## Introduction

Hereditary spastic paraplegias (HSPs) are a group of heterogenous genetic neurodegenerative diseases characterized by muscle weakness and spasticity that lead to a gradual loss of the ability to walk (Shribman et al., 2019). These diseases are caused by length-dependent degeneration of the axons of the corticospinal tract and are classified into pure or complex forms, the latter of which have pyramidal signs accompanied by other neurological or systemic abnormalities (Harding, 1983; DeLuca et al., 2004; Schüle et al., 2016). More than 88 *loci* were associated with HSPs, with SPG4 being the most common subtype worldwide, representing 50% of cases with autosomal dominant inheritance in Brazil (Shribman et al., 2019; Fussiger et al., 2022).

Although significant advances have been made in the understanding of the molecular biology of HSP (Elsayed et al., 2021) and in the development of novel therapeutic strategies for genetic diseases such as gene replacement, editing, and RNA-based therapies (Kulkarni et al., 2021), the regulatory approval of disease-modifying treatments for HSP will likely be hampered by the lack of fit-for-purpose outcome measures to evaluate efficacy in clinical trials. Clinical outcome assessment (COA) is a measure that describes or reflects how a patient feels, functions, or survives (FDA, 2022), with the clinician-reported outcome (ClinRO) Spastic Paraplegia Rating Scale (SPRS) being the most studied COA in HSP. While in the few longitudinal studies, performed in small and heterogeneous samples, there were conflicting results on SPRS sensitivity to change (Cubillos-Arcila et al., 2022; Amprosi et al., 2023), larger sample cross-sectional studies consistently showed very slow disease progressions (Schöls et al., 2017; Giordani et al., 2021; Rossi et al., 2022).

Performance outcomes (PerFOs), another type of COA, theoretically offer higher sensitivity to change compared to ClinROs, owing to their quantitative and continuous nature. This could potentially provide an advantage as a trial endpoint for diseases with slow progression, such as HSPs. In a recent study, we demonstrated satisfactory discriminatory, face, and construct validity of the PerFO: 10-meter walking test (10MWT) and Timed-Up and Go (TUG) at self-selected and maximal walking speeds, as well as the 6-min walking test (6MWT) and the locomotor rehabilitation index (LRI) in HSP patients when compared to matched controls (Cubillos-Arcila et al., 2022). Contrary to what would be expected, there was no deterioration in PerFOs after 1.5 years of follow-up. Now, we aimed to describe the long-term progression of ClinROs and PerFOs in the same HSP cohort after 3 (from 1.5 to 4.5 years) and 4.5 years of follow-up.

## Materials and methods

### Design and ethics

This study is the long-term continuation of a prospective cohort (Cubillos-Arcila et al., 2022), which was approved by the Ethics in Research Committee of HCPA (GPPG-HCPA-2017-0341), following the Declaration of Helsinki. Informed written consent was obtained from all subjects or their guardians.

### Subjects

**Figure 1:**
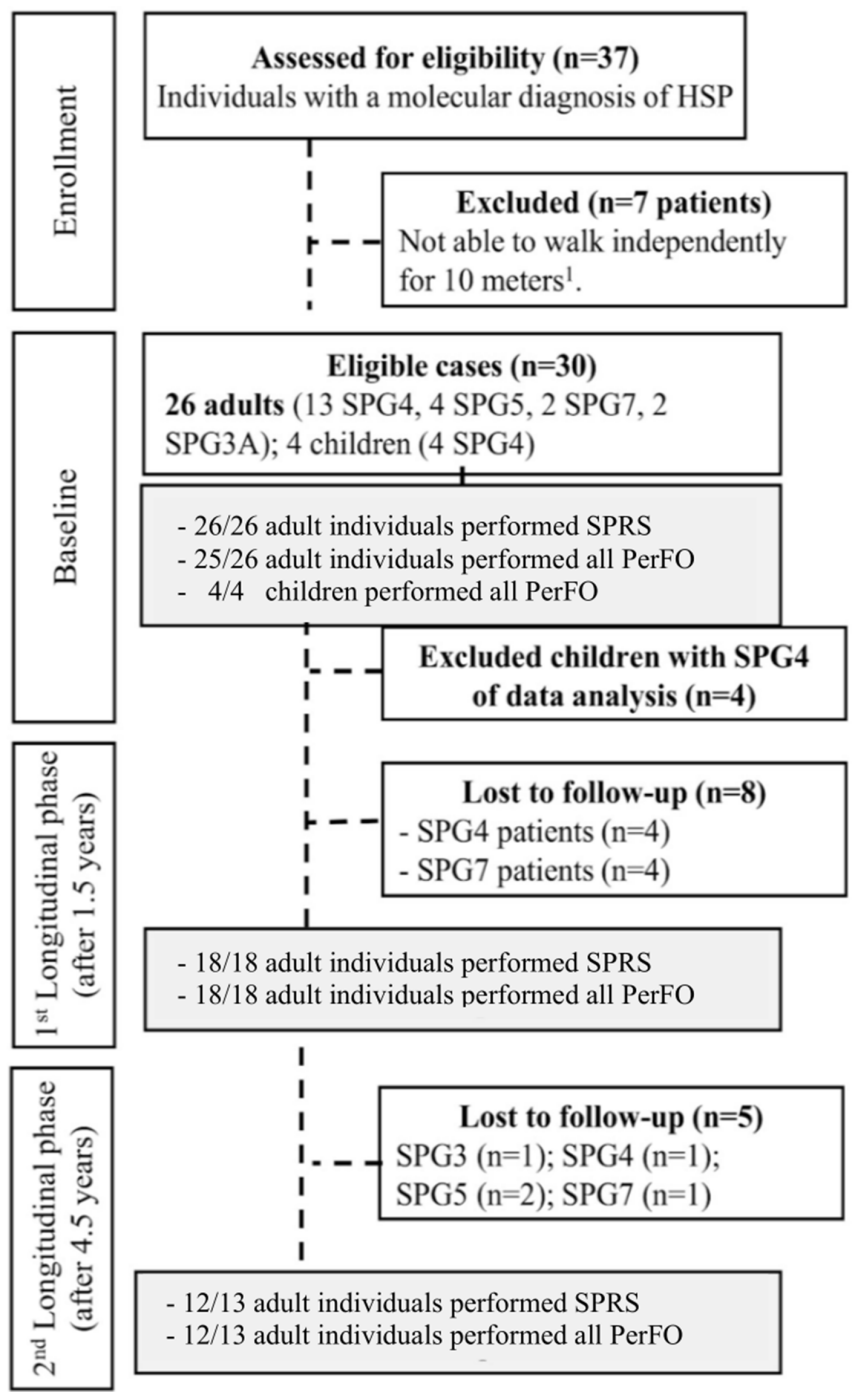
Study flowchart. HSP, hereditary spastic paraplegias; PerFO, performance outcome; 1, Canes, crutches or walkers were allowed; 2, A single patient did not perform PerFO in the baseline evaluation; and 3, A single patient did not perform SPRS in the 4.5-year evaluation.

Twenty-six adult patients with HSP were eligible for the study (15 with SPG4, 5 with SPG7, 4 with SPG5, and 2 with SPG3A). All subjects were assessed at baseline, 18 after 1.5 years, and 13 after 4.5 years of follow-up (Figure 1). They were recruited from January 2018 to July 2018 at a single center, the Neurogenetics outpatient clinic of the Hospital de Clínicas de Porto Alegre (HCPA).

In brief, inclusion criteria were a previously defined genetic diagnosis of HSP, being able to walk independently for at least 10 meters, with or without walking aids, and being on stable physical therapy or antispastic medication for 6 months before study entry. The analysis exclusively involved adults, as supported by findings from the 1.5-year follow-up of the cohort. Unlike adults, children with HSP demonstrated PerFO improvements over time, possibly due to the biomechanical impacts of growth, including increased height and lower limb length (Cubillos-Arcila et al., 2022). Moreover, validity studies of SPRS for this particular population had not been previously performed. Further study design information is available elsewhere (Cubillos-Arcila et al., 2022).

### Procedures and clinical outcome assessments

Disease severity was evaluated by the Brazilian Portuguese version of SPRS (range: 0–52, crescent in severity); we also performed an exploratory analysis of the motor-SPRS (mSPRS), excluding non-motor items 12 and 13, which are related to pain and sphincter control (range: 0–44). The full version of the scale can be found elsewhere (Schule et al., 2006; Servelhere et al., 2016). Disease stage was classified as (0) asymptomatic; (1) absence of functional handicap, but the presence of physical signs at examination (slight gait stiffness); (2) mild gait stiffness, unlimited walking, still able to run; (3) moderate gait stiffness, limited walking without aid, unable to run; (4) moderate to severe gait stiffness, able to walk only with aid (orthosis); (5) unable to walk, wheelchair-bound (Dürr et al., 1996). PerFO of gait included three tests in the following order of application: (a) 10MWT for the measurement of self-selected walking (SSWS) and maximal walking speeds (MWSs) (Watson, 2002; Adell et al., 2012); (b) TUG at SSWS and MWS for quantifying functional mobility (Podsiadlo and Richardson, 1991); and (c) 6-min walking test (6MWT) to assess aerobic capacity and endurance (ATS, 2017). Finally, the locomotor rehabilitation index (LRI) was calculated (Peyré-Tartaruga and Monteiro, 2016).

The Clinical Global Impression-Improvement Scale (CGI) is a 7-point patient-reported outcome designed to assess the extent of improvement or worsening of a patient’s condition compared to a baseline state with a specific treatment or over a period of natural progression (Busner and Targum, 2007). Instances where patients reported very little, much, or minimal improvements or worsening were consolidated into improvement or worsening categories. This CGI categorization was employed as an anchor to establish the minimum clinically important differences (MCIDs) of the studied COAs, which represent the smallest change in the outcomes that patients perceive as either beneficial or detrimental (Jaeschke et al., 1989). Additional details of the study procedures can be found elsewhere (Cubillos-Arcila et al., 2022).

### Statistical analysis

Statistical tests were selected according to the distribution of data given by quantile–quantile plot (Q–Q plot) and Shapiro–Wilk test. Only SPRS, mSPRS, 6MWT, and LRI presented normal distributions. Variables that did not have a normal distribution were log-transformed, except for 10MWT-MWS, which was Box-Cox transformed, for the analyses.

For assessing COA progression, two strategies were designed, one that modeled the study duration (follow-up time) and the other that modeled progression according to an individual’s disease duration at the moment of the different evaluations. For the study follow-up time analysis, we applied a generalized estimated equation (GEE) with the Bonferroni post-hoc test. For the progression analysis with disease duration as the timeframe, a mixed model, considering random effects for each person and for time, was performed, in which disease duration was considered as a continuous covariate, estimated as the difference between the participant’s age at evaluations and the reported onset. Data were analyzed for the total HSP sample and for the SPG4 subgroup. Considering the slower progression of childhood-onset SPG4 compared to adult-onset SPG4 (Schüle et al., 2016; Parodi et al., 2018; Rossi et al., 2022), we also performed a subgroup analysis only with adult-onset cases. Correlations between SPRS and PerFo progression scores were performed with Spearman’s correlation tests using the mean of the difference between the last and baseline evaluations.

Sample size estimations were performed with the observed progressions of the different COAs using the disease duration model and hypothetical intervention effects that could reduce progression rates or ameliorate baseline scores. This analysis was performed in steps of 25% with power set at 0.8 and α at 0.05 with the longpower R package.

The ability to detect clinically important changes was assessed as the area under the receiver operating characteristic (ROC) curve of each COA score change against worse, stable, or better according to the clinical global impression-improvement scale (CGI) as an external criterion. The upper limit of the 95% confidence interval (CI) of change in the stable subjects was used as a conservative estimate of minimal clinically important differences (MCIDs).

The threshold for statistical significance was at a value of p of <0.05 (95% confidence interval). GEE was estimated in SPSS v18, and mixed models were estimated in R 4.2.0 with the lme4 package.

## Results

The main clinical and demographic characteristics of the HSP patients and SPG4 subgroup are shown in Table 1. Progression analysis according to the study follow-up time and the individual’s disease duration will be presented separately.

**Table 1:** Main clinical and demographic characteristics.

	Baseline		1.5 years		4.5 years	
	HSP (*n* = 26)	SPG4 (*n* = 15)	HSP (*n* = 18)	SPG4 (*n* = 11)	HSP (*n* = 13)	SPG4 (*n* = 10)
Female	14/26 (65%)	9/15 (40%)	10/18 (55%)	6/11 (60%)	6/13 (50%)	5/10 (50%)
Age—years	46 (12)	48.6 (3.2)	50 (12)	52 (3.4)	50 (12)	53.4 (3.5)
Leg length—cm	88 (6.5)	88.7 (0.9)	88 (6.5)	88 (6.5)	88 (6.5)	88 (6.5)
SPRS	18 (1.8)	17 (2.5)	19 (2.4)	19 (3.1)	17 (2.6)	17 (3.1)
mSPRS	17 (1.5)	16 (2.3)	16 (2.3)	16 (2.8)	16 (2.4)	14 (2.9)
Disease duration—years	17 (10)	16 (2.3)	17 (10)	16.5 (2.9)	17 (10)	18.8 (3.3)
Age at onset—years	30 (17)	34.5 (4.9)	30 (4.2)	35 (5.5)	32 (5.1)	35 (6.1)
Walking-aid assistance	15/26 (58%)	10/15 (67%)	10/18 (56%)	7/11 (64%)	7/13 (54%)	6/10 (60%)
Clinical form	24/26 pure (92%)	100% pure	16/18 pure (88%)	100% pure	100% pure	100% pure
Disease stage	1–2 (8%)	1–1 (6%)	1–2 (11%)	1–1 (9%)	1–0 (0%)	1–0 (0%)
	2–3 (12%)	2–3 (20%)	2–3 (17%)	2–3 (27%)	2–1 (7%)	2–1 (10%)
	3–8 (30%)	3–4 (27%)	3–4 (22%)	3–1 (18%)	3–5 (39%)	3–3 (30%)
	4–13 (50%)	4–7 (47%)	4–9 (50%)	4–6 (46%)	4–7 (54%)	4–6 (60%)
Symptomatic treatment						
Botulinum toxin	2/26 (8%)	1/15 (7%)	1/18 (6%)	1/11 (9%)	1/13 (8%)	1/10 (10%)
Baclofen/Tizanidine	4/26 (15%)	1/15 (7%)	6/18 (33%)	4/11 (36%)	5/13 (39%)	2/13 (15%)
Physical therapy	13/26 (50%)	6/15 (40%)	12/18 (67%)	6/11 (55%)	8/13 (62%)	4/10 (40%)

Data are shown in frequency (percentage) and mean (standard deviation). cm, centimeters; HSP, hereditary spastic paraplegias; SPRS, Spastic Paraplegia Rating Scale; mSPRS, Motor Spastic Paraplegia Rating Scale.

### COA progressions according to the study follow-up time

**Figure 2:**
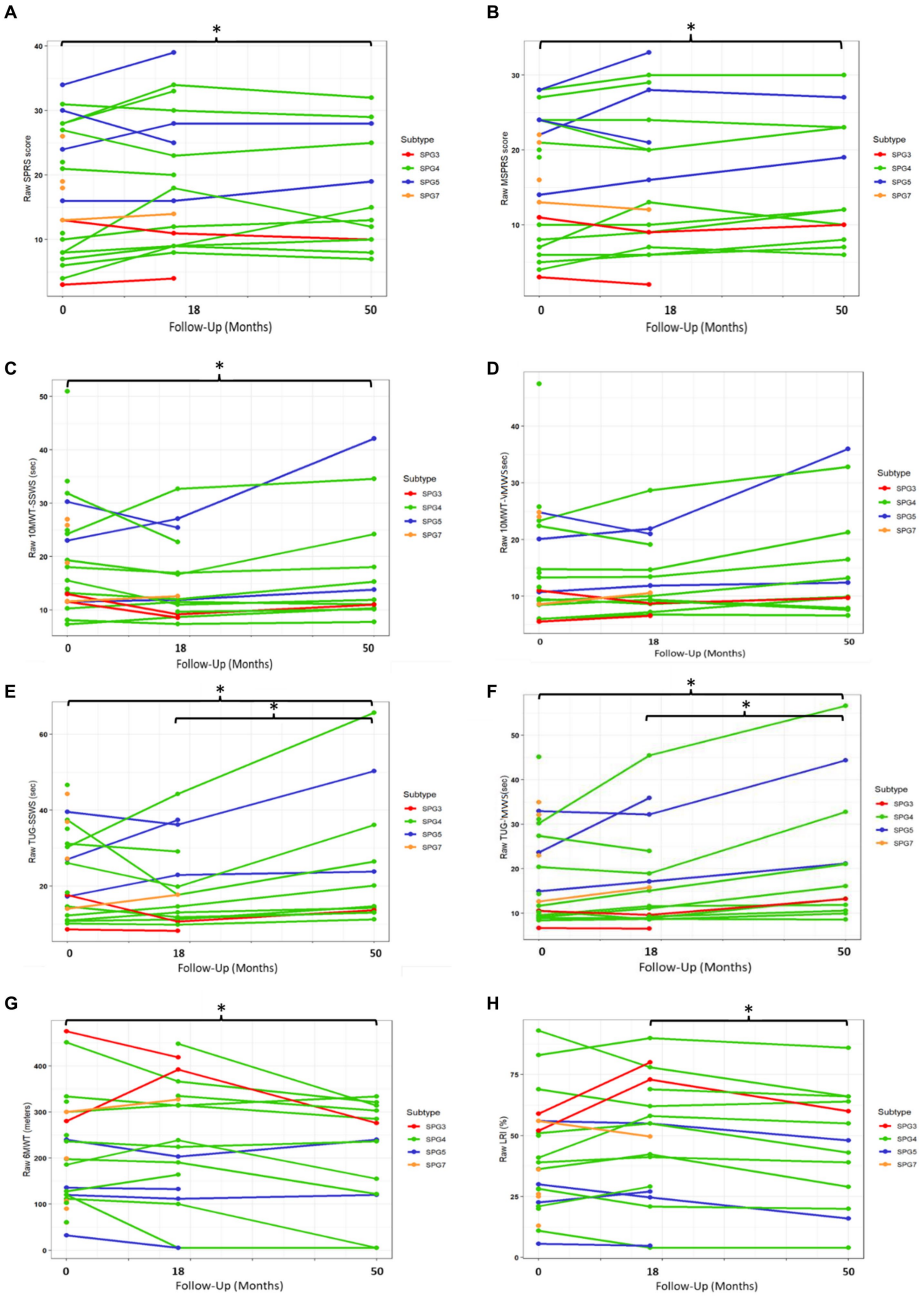
Progression of ClinROs and PerFOs according to the study follow-up time in the overall HSP group. SPRS, Spastic Paraplegia Rating Scale; mSPRS, Motor Spastic Paraplegia Rating Scale; 10MWT-SSWS, 10-meter walking test at self-selected speed; 10MWT-MWS (s), 10-meter walking test at maximal speeds; TUG-SSWS, Timed-Up and Go at self-selected walking speed; TUG-MWS, Timed-Up and Go test at maximal walking speed; LRI: locomotor rehabilitation index (%); 6MWT: 6-min walking test. *p < 0.05.

In the overall HSP sample, there was no significant progression of all studied COAs from baseline to 1.5 years (*p* > 0.05 for all comparisons, Table 2 and Figure 2). From 1.5 to 4.5 years (3-year interval), it was possible to detect the progression of the PerFOs: TUG-SWSS, TUG-MWS, 10MWT-SWSS, and LRI (*p* < 0.05 for all comparisons, Table 2 and Figure 2). Finally, from baseline to 4.5 years, there were significant progressions of the ClinROs: SPRS and mSPRS, and of the PerFOs: 10MWT-SWSS, 6MWT, TUG-SWSS, and TUG-MWS (p < 0.05 for all comparisons, Table 2 and Figure 2). There was no significant progression of 10MWT-MWS during the study follow-up. SPG4 subgroup results were similar to the overall HSPs, except for TUG-SWSS, which differed only between baseline and 4.5 years (Table 2 and Supplementary Figure S1).

**Table 2:** Progression of clinical outcome assessments according to the study follow-up time in the overall HSP group and in the SPG4 subgroup.

HSP group						SPG4 group			
COA	Assessment	Mean	95% CI	*p* ^1^	Statistical Difference (*)	Mean	95% CI	*p* ^1^	Statistical difference (*)
SPRS	Baseline	18.3	14.8–21.9	0.030	A	17.27	12.5–22.0	0.018	A
	1.5 years	20	16.5–23.6		AB	19.7	15.1–24.3		AB
	4.5 years	20.3	16.9–23.7		B	19.7	15.5–23.9		B
mSPRS	Baseline	16.6	13.6–19.7	0.002	A	16	11.5–20.4	0.005	A
	1.5 years	17.5	14.2–20.9		AB	16.9	12.7–21.1		AB
	4.5 years	18.4	15.3–21.6		B	17.6	13.6–21.7		B
10MWT-SSWS—s	Baseline	17.3	14.1–21.3	<0.001	AB	17.2	12.8–23	0.006	AB
	1.5 years	16	13–19.7		A	16.4	12.2–21.8		A
	4.5 years	19.4	15.1–24.8		B	18.7	13.7–25.5		B
10MWT-MWS—s	Baseline	12.4	10–16	ns	A	12.2	9.4–16.9	ns	A
	1.5 years	11.1	9.2–13.6		A	10.8	8.7–14		A
	4.5 years	11.9	9.4–16.1		A	11.3	8.7–15.6		A
TUG-SSWS—s	Baseline	20.3	16.3–25.3	<0.001	A	18.7	14–24.9	<0.001	AB
	1.5 years	19.5	15.4–24.		A	17.7	13.5–23.3		A
	4.5 years	25	19.1–32.8		B	23.5	16.8–32.9		B
TUG-MWS—s	Baseline	16.1	12.8–20.3	<0.001	A	14.7	10.6–19.8	<0.001	A
	1.5 years	17.6	13.6–22.8		A	15.9	11.6–22		A
	4.5 years	22.6	16.9–29.9		B	20.6	14.2–29.7		B
6MWT—m	Baseline	218.4	168.4–268.5	0.018	A	236.8	177.9–295.5	<0.001	A
	1.5 years	209.1	157.5–260.6		AB	226.5	161.5–291.5		A
	4.5 years	175.2	126.6–223.8		B	178.5	161.5–291.5		B
LRI—%	Baseline	40	31.5–48.5	<0.001	AB	44	31.7–56.3	0.008	AB
	1.5 years	42.3	32.9–51.8		A	45	32.6–56.8		A
	4.5 years	35.8	26.6–45.1		B	40	27.5–51.4		B

Data are shown as mean and 95% confidence intervals. Variables that did not have normal distribution were log-transformed, except for 10MWT-MWS which was Box-Cox transformed, for the analyses and back-transformed to raw values to be presented in the table. m, meters; s, seconds; COA, clinical outcome assessments; HSP, hereditary spastic paraplegia; LRI, locomotor rehabilitation index (%): percentage; 6MWT, 6-min walking test; SPRS, Spastic Paraplegia Rating Scale; mSPRS, Motor Spastic Paraplegia Rating Scale; 10MWT-SSWS, 10-meter walking test at self-selected speed; 10MWT-MWS (s), 10-meter walking test at maximal speeds; TUG-SSWS, Timed-Up and Go test at self-selected walking speed; TUG-MWS, Timed-Up and Go test at maximal walking speed. ^1^GEE test; Bonferroni post-hoc test. *The presence of different capital letters between the evaluation points indicates the presence of statistically significant differences. For instance, in the case of SPRS, baseline represented by A and 4.5 years by B indicate differences between these times, while the representation AB in 1.5 years indicates that there is no difference between this assessment and baseline or 4.5-year assessments.

There were moderate direct correlations between mean SPRS and mSPRS progressions during the whole study follow-up with mean 10MWT-SSWS and 10MWT-MWS progressions for the overall HSP group (Rho = 0.548–0.679, *p* < 0.05 for all comparisons). The studied ClinRO progressions did not correlate with TUG, 6MWT, or LRI progressions (Table 3).

**Table 3:** Correlations between clinician-reported and performance outcomes during 4.5 years of follow-up in the overall HSP group.

COA		Delta 10MWT-SSWS	Delta 10MWT-MWS	Delta TUG-SSWS	Delta TUG-MWS	Delta 6MWT	Delta LRI
Delta SPRS	Rho	0.618**	0.679**	0.477	0.242	−0.473	−0.438
	*p*	0.008	0.003	0.053	0.350	0.064	0.079
Delta mSPRS	Rho	0.638**	0.548*	0.366	0.219	−0.298	−0.477
	*p*	0.006	0.023	0.148	0.399	0.262	0.053

SPRS, Spastic Paraplegia Rating Scale; mSPRS, Motor Spastic Paraplegia Rating Scale; COA, clinical outcome assessments; 10MWT-SSWS, 10-meter walking test at self-selected speed; 10MWT-MWS (s), 10-meter walking test at maximal speed; TUG-SSWS, Timed-Up and Go test at self-selected walking speed; TUG-MWS, Timed-Up and Go test at maximal walking speed; 6MWT, 6-min walking test; %, percentage; LRI, locomotor rehabilitation index. *p < 0.05, **p < 0.01.

### Annual COA progressions modeled by disease duration

**Figure 3:**
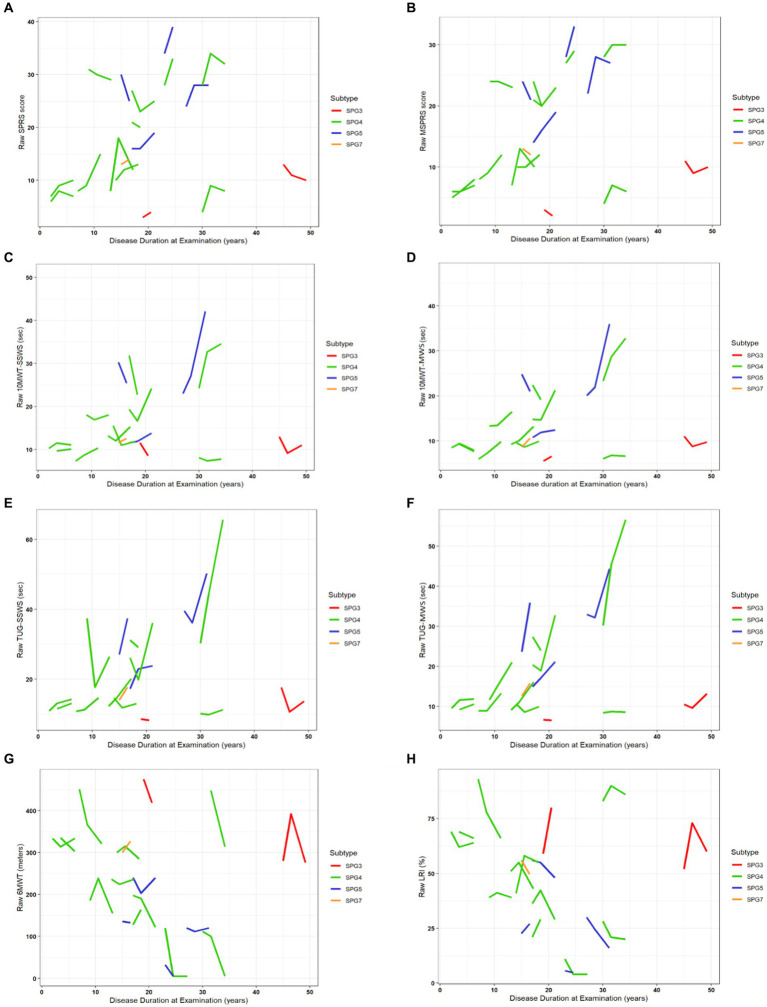
Progression of ClinROs and PerFOs modeled by disease duration in the overall HSP group. **(A–H)** indicate the results for the different clinical outcome assessments. SPRS, Spastic Paraplegia Rating Scale; mSPRS, Motor Spastic Paraplegia Rating Scale; 10MWT-SSWS, 10-meter walking test at self-selected speed; 10MWT-MWS (s), 10-meter walking test at maximal speeds; TUG-SSWS, Timed-Up and Go at self-selected walking speed; TUG-MWS, Timed-Up and Go test at maximal walking speed; LRI, locomotor rehabilitation index (%); 6MWT, 6-min walking test.

**Figure 4:**
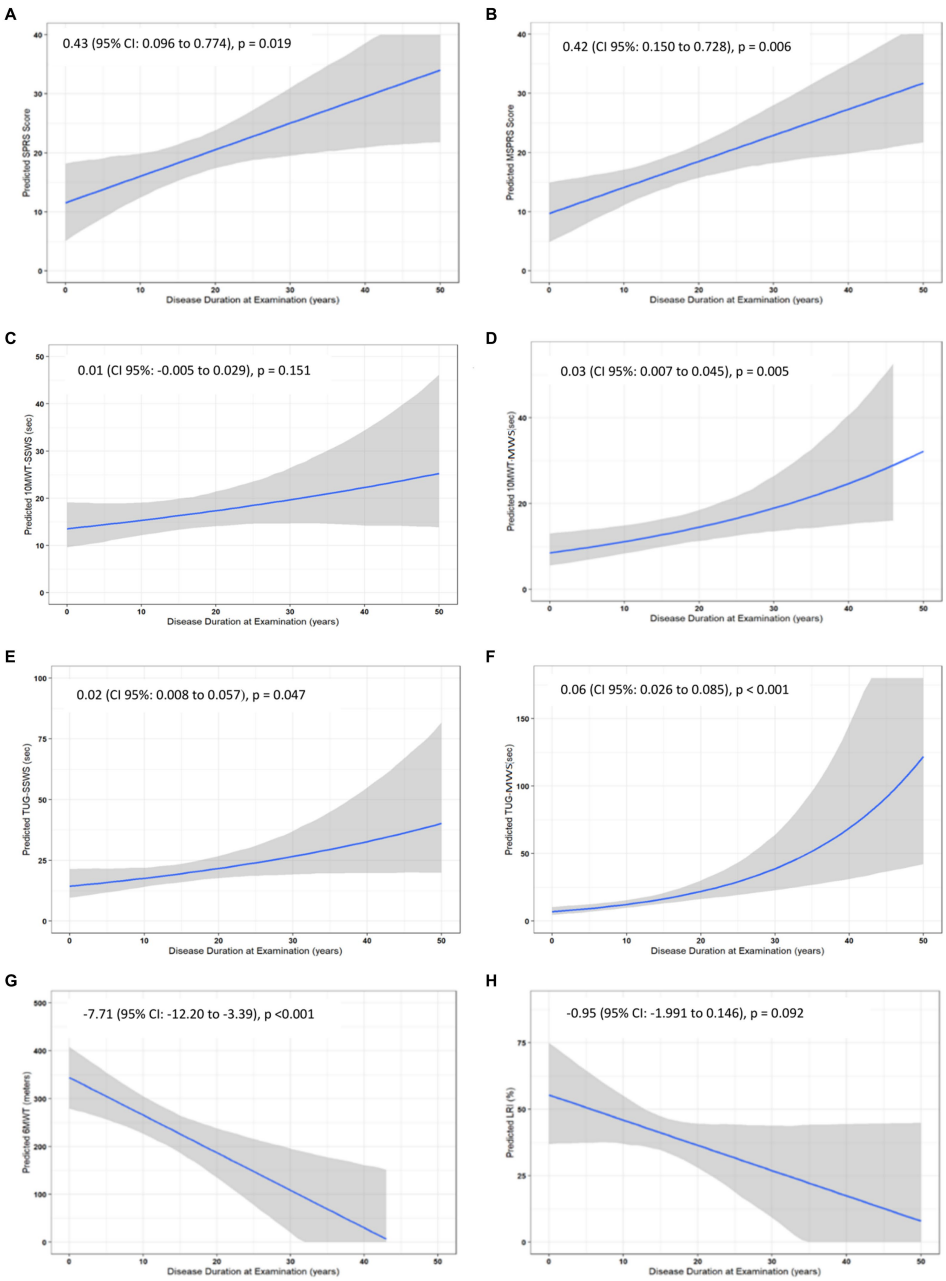
Annual progression of clinical outcome assessments in the overall HSP group. **(A–H)** indicate the results for the different clinical outcome assessments. Variables that did not have a normal distribution were log-transformed, except for 10MWT-MWS which was Box-Cox transformed, for the analyses and back-transformed to raw values. SPRS, Spastic Paraplegia Rating Scale; mSPRS, Motor Spastic Paraplegia Rating Scale; 10MWT-SSWS, 10-meter walking test at self-selected speed; 10MWT-MWS (s), 10-meter walking test at maximal speeds; TUG-SSWS, Timed-Up and Go at self-selected walking speed; TUG-MWS, Timed-Up and Go test at maximal walking speed; LRI, locomotor rehabilitation index (%); 6MWT, 6-min walking test.

There were statistically significant annual progressions for the ClinROs and PerFOs, with the exception of the LRI and 10MWT-SSWS, in the analysis modeled by disease duration in the overall HSP group (Supplementary Table S1 and Figures 3, 4). For example, the mean annual progression of SPRS was 0.43 points (95% CI: 0.096–0.774, *p* = 0.019, Figure 4A), and the mean annual progression of TUG-MWS was 0.06 s (95% CI: 0.026–0.085; *p* < 0.001, Figure 4F). The results were similar for the SPG4 subgroup (Supplementary Table S1), whereas in the analysis of the adult-onset subgroup, all COAs showed statistically significant annual progressions (Supplementary Table S1).

### Minimal clinically important differences of COAs

**Figure 5:**
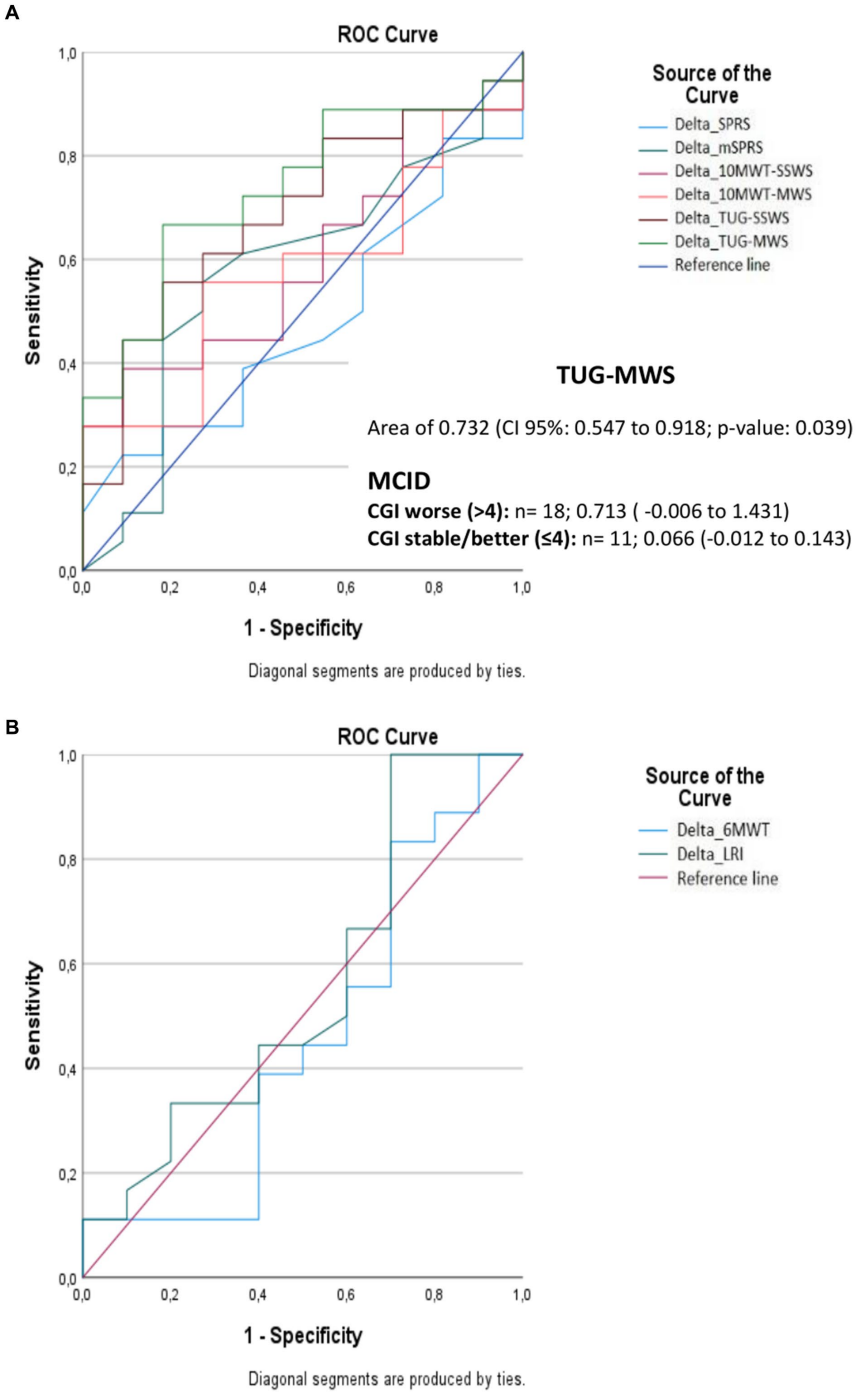
Area under the curve for detecting minimal clinically important differences in clinical outcome assessments according to the clinical global impression of change. **(A,B)** indicate the results for the different clinical outcome assessments. SPRS, Spastic Paraplegia Rating Scale; mSPRS, Motor Spastic Paraplegia Rating Scale; 10MWT-SSWS, 10-meter walking test at self-selected speed; 10MWT-MWS (s), 10-meter walking test at maximal speeds; TUG-SSWS, Timed-Up and Go at self-selected walking speed; TUG-MWS, Timed- Up and Go test at maximal walking speed; LRI, locomotor rehabilitation index (%); 6MWT, 6-min walking test; ROC, receiver operating characteristic curve; MCID, minimal clinically important differences; CGI, clinical global impression.

TUG-MWS was the only COA that accurately discriminated subjects with worse CGI (>4) from subjects with stable/better CGI (≤4), with an area under the curve of 0.732 (95% CI: 0.547–0.918; *p*-value: 0.039) The progression of TUG-MWS in subjects that noticed worsening according to CGI (*n* = 18) was 0.713 s (95% CI: −0.006 to 1.431), and its progression in stable/better subjects (*N* = 11) was 0.07 s (95% CI: −0.012 to 0.143) (Figure 5 and Supplementary Table S2). According to this, the MCID for TUG-MWS would be 0.15 s.

### Sample size estimations for future clinical trials

**Figure 6:**
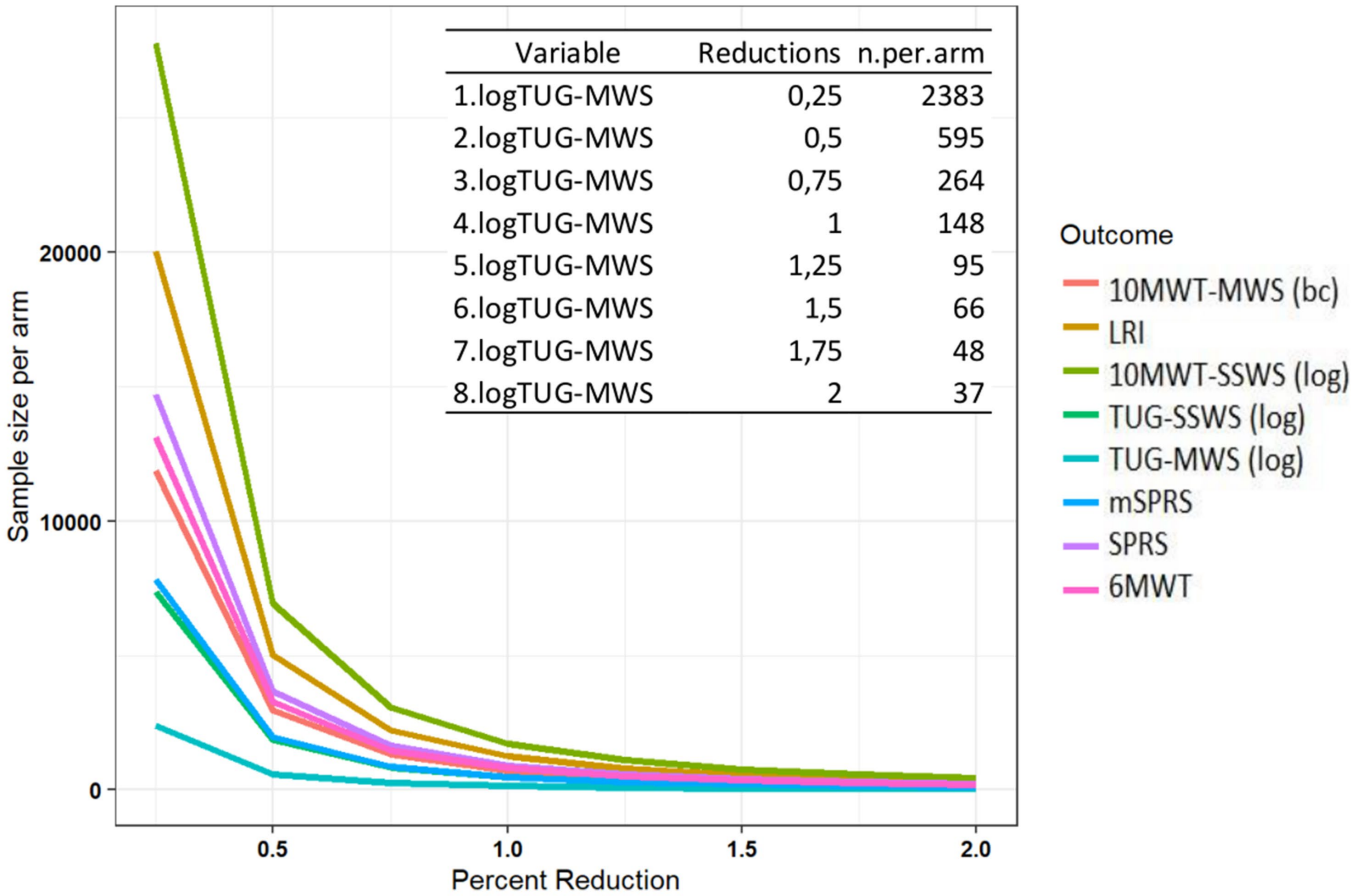
Sample size estimation for future clinical trials using the disease duration model. SPRS, Spastic Paraplegia Rating Scale; mSPRS, Motor Spastic Paraplegia Rating Scale; 10MWT-SSWS, 10-meter walking test at self-selected speed; 10MWT-MWS (s), 10-meter walking test at maximal speeds; TUG-SSWS, Timed-Up and Go at self-selected walking speed; TUG-MWS, Timed-Up and Go test at maximal walking speed; 6MWT, 6-min walking test; (%), Percentage; LRI, locomotor rehabilitation index; Log, Logarithm; bc, Box-Cox.

Considering that statistically significant progressions in COAs were identified between 3 and 4.5 years of follow-up within the study’s duration model, and acknowledging that these timeframes might be excessively lengthy for phase 2 clinical trials, we decided to provide sample size estimations with progressions based on the disease duration model. Sample size estimations with different effect sizes on the slope of the studied COAs are presented in Supplementary Table S3. TUG-MWS was the COA that would require the smallest sample size for a trial, and 579 patients per study arm for a treatment that would reduce the disease progression slope by 50%, 144 patients/arm for a treatment that stops the disease progression, and 36 patients/arm for a treatment that would lead to improvement in the same range of the annual progression (Figure 6 and Supplementary Table S3).

## Discussion

According to the FDA, clinical outcome assessments (COAs) must be developed or modified to be fit-for-purpose and patient-focused (FDA, 2022). In this study, long-term longitudinal data that allowed evaluating the concepts of interest of disease progression (sensitivity to change) in hereditary spastic paraplegias of two types of COAs, two ClinROs: the SPRS, and a reduced version of the scale, the mSPRS; and 6 PerFOs, 10-meter walking test (10MWT) and Timed-Up and Go (TUG), at self-selected and maximal walking speeds, a 6-min walking test (6MWT), and the locomotor rehabilitation index (LRI), were provided. During the 3-year interval between the second and third assessments, significant progressions were only found in PerFOs, indicating greater sensitivity to change in this type of outcome, and over the 4.5 years of follow-up, both PerFOs and ClinROs displayed significant progressions. Through statistical modeling, we scrutinized the trajectory of COA progression based on the disease duration at the time of the assessments. This enabled the estimation of annual progression rates for the different outcomes and the estimation of sample sizes needed for forthcoming clinical trials involving interventions with varying effect sizes.

### Clinician-reported outcome—spastic paraplegia rating scale

Spastic Paraplegia Rating Scale (SPRS) is a clinical rating scale developed by a German task force with the aim of evaluating disease severity of pure and complex HSP (Schule et al., 2006). Published in 2006, SPRS is the most widely used COA in the study of HSPs, consisting of 13 items that assess the following functions/physical examination findings/symptoms: walking distance, quality and speed of gait/run, quality and speed of climbing up and down stairs, arising from a chair, spasticity in hip adductor and knee extensor muscles, weakness of hip abduction and foot dorsiflexion, contractures of lower limbs, pain due to HSP-related symptoms, and bladder and bowel function. SPRS had high internal consistency and interrater agreement, with adequate construct and criterion validity, for both pure and complex HSP (Schule et al., 2006). Proper sensitivity to change analysis of SPRS was not assessed in the validation study. Recently, we did not identify statistically significant progression of SPRS and mSPRS after 1.5 years of follow-up in 18 patients with HSP (10 with SPG4, Cubillos-Arcila et al., 2022). With the study continuing for 4.5 years in total, a significant 2-point difference was found between baseline and the last SPRS assessment (2.43 points in the subgroup with SPG4) and 1.8 points for mSPRS (1.6 points in the subgroup with SPG4). The annual progression of SPRS was estimated at 0.43 points when modeled by disease duration and considering the 57 assessments performed during the study. SPRS progression was not able to differentiate patients with a worse compared to a stable/better impression of change in CGI, and it was not possible to estimate its MCID. Different studies have evaluated the progression over time of SPRS in HSPs. Cross-sectional disease progression (SPRS score/disease duration) was between 0.83 and 1.7 points per year in a large case series of 278 patients with SPG4 (Rossi et al., 2022), ranging from 1.08 points per year for SPG4 to 1.37 points per year for SPG11 in a large series of childhood-onset HSP (Giordani et al., 2021), and was 0.56 points per year in 34 cases from 28 families with SPG5 (Schöls et al., 2017). Progression in longitudinal studies has been estimated at 0.5 points per year in a subsample of 28 patients with SPG4 (Rossi et al., 2022), 0.8 points per year in a subset of 21 patients with SPG5 (Schöls et al., 2017), significant worsening after 14 months in a sub-cohort of 11 patients with complex HSP (Regensburger et al., 2022), and 1.17 points in a subsample of 30 HSP patients (most with complex HSP and many without genetic confirmation of the diagnosis, Amprosi et al., 2023). The sensitivity to change in SPRS was also evaluated in the context of clinical trials. In the only double-blind randomized clinical trial found, there were no differences in SPRS after 8 weeks of intramuscular botulinum toxin A (BoNT-A) treatment on *adductor magnu*s and *triceps surae* when compared to placebo in 54 patients with a genetic diagnosis of HSP (Diniz de Lima et al., 2021). Open-label trials found different treatment responsiveness to SPRS. The median SPRS was 1.5 points lower after 3 months of BoNT-A treatment and intensive physical therapy in different spastic muscles under electromyographic guidance in 18 Italian HSP patients (11 with genetic diagnosis, Paparella et al., 2020), and the mean SPRS was reduced by 1.9 points after 2 weeks of dalfampridine treatment in 12 HSP patients (Béreau et al., 2015). Considering all these data, SPRS is capable of detecting disease progression; however, there is considerable variability in the estimates of its rate of progression, which can be explained by the different contexts in which the scale was used and by the small sample sizes of the prospective longitudinal studies. Conversely, the scale’s responsiveness to treatments remains less clear. This is highlighted by the fact that while sole double-blind randomized trial utilizing SPRS as an outcome yielded negative results (Diniz de Lima et al., 2021), the two open-label studies (Béreau et al., 2015; Paparella et al., 2020) demonstrated improvements in short periods of time and with large effect sizes.

Despite the previously performed validation and recent data indicating sensitivity to changes in SPRS, it is not clear whether the original version of the scale meets the requirements of regulatory agencies for COAs. In the present study, SPRS was considered a ClinRO; however, the items related to walking distance, pain due to HSP-related symptoms, and bladder and bowel function would be better qualified as patient-reported outcomes (PROs), as well as the items that evaluate the speed of gait/run and of climbing stairs. The concept of interest (COI) evaluated with SPRS is also not clear. The scale was developed to assess disease severity, but this concept is too vague or not completely achieved by the scale, as the disease can affect multiple neurological systems, and cognitive, somatosensory, among other domains are not addressed by the instrument. Some of the scoring responses of the SPRS might be arbitrary and likely have an important influence on the scale’s sensitivity to change. For instance, consider the case of the walking distance item, where a score of 2 signifies distances less than 500 m and a score of 3 corresponds to distances less than 10 m. This structure implies that a patient who initially walked 400 m and then experienced a decline of 300 m within a year would not exhibit a change in score. Another concern relates to the precision and comprehensiveness of certain item descriptions, such as the maximum gait speed item, which can be interpreted as having to be assessed while running or walking or as having to be assessed only while walking, due to potential ambiguity in the provided details. All these points may explain the variability of results found by different groups and indicate that adapted versions of the scale should be developed for different COIs, including the disease progression, the focus of the present study, and for different COUs, including separate studies for specific subtypes, early disease and moderate/advanced disease. Additionally, it will be necessary to anchor SPRS with PROs or qualitative views of patients to define which changes represent relevant effects for patients so that the scale can be used as an endpoint recognized by regulatory agencies for future clinical trials.

### Performance outcomes

In the cross-sectional phase of our previous study, we validated some of the main psychometric characteristics of gait PerFOs for HSPs (Cubillos-Arcila et al., 2022), which were confirmed by another recent study, in which 10MWT at SSWS, TUG, and 2-min walking test (2MWT) strongly correlated with the fear of falling and moderately correlated with the physical component of quality of life in 22 patients with HSP (Gaßner et al., 2021). However, in the 18-month longitudinal phase, we did not observe significant progressions in PerFOs, lacking confirmation of their sensitivity to change (Cubillos-Arcila et al., 2022). In the current analysis, with a longer follow-up period, it was possible to carry out additional comparisons of 3 (between 1.5 and 4.5 years) and 4.5 years (between baseline and 4.5 years), making it possible to define the progression of outcomes. Sensitivity to change in the study’s follow-up analysis was greater for TUG-SWSS, TUG-MWS, 10MWT-SWSS, and LRI than for SPRS, with these PerFOs capturing significant changes over a 3-year period (from 1.5 to 4.5 years), while SPRS showed significant progression only after 4.5 years of follow-up, confirming the premise that PerFOs in general are more sensitive to change than semi-quantitative instruments such as SPRS.

#### 10-meter walking test and locomotor rehabilitation index

After 3 years of follow-up in the overall HSP group, significant differences were found only for 10MWT at self-selected walking speed (SSWS), whereas when modeled by disease duration, significant annual progression was only found for 10MWT at maximum walking speed (MWS), with an increment of 0.03 s/year. Both test conditions showed significant annual progressions in the adult-onset subgroup. 10MWT progression in both conditions was not able to differentiate patients with a worse compared to a stable/better impression of change in CGI, and it was not possible to estimate 10MWT MCID. We found only a single observational study that evaluated 10MWT-MWS progression in patients with HSPs, showing statistically significant progression in longitudinal follow-up gait analysis in a sub-cohort of 11 patients with complex HSP after 14 months (Regensburger et al., 2022). At least four studies used 10MWT as a clinical trial endpoint for HSP. In the only randomized clinical trial, Diniz de Lima et al. used the speed of 10MWT-MWS after 8 weeks of treatment with BoNT-A as the primary endpoint and 10MWT-SSWS as a secondary endpoint, and the results were negative (Diniz de Lima et al., 2021). Two open-label studies evaluated the response of 10MWT with the use of BoNT-A associated with rehabilitation measures. A Dutch study evaluated the effectiveness of injections in each *triceps surae* (Dysport^®^, 500–750 MU) followed by daily stretching exercises (18 weeks) in 15 subjects with pure hereditary spastic paraplegia, and found an increase in speed in 10MWT-SSWS of 0.9 m/s and 0.98 m/s after 4 and 18 weeks, respectively (de Niet et al., 2015); interestingly, the mean velocity at MWS did not change with treatment, remaining constant across all time points. In the study by Paparella et al., there was a reduction in the median of the 10MWT-SSWS of 1.9 s after 1 month and 2 s after 3 months of treatment compared to the baseline, and the test was not performed in MWS (Paparella et al., 2020). Finally, the open-label study that evaluated the efficacy of dalfampridine in HSP showed no treatment response in the Timed-25-Foot Walk (which represents 7.62 meters) at MWS after 2 weeks (Béreau et al., 2015). We found moderate correlations between 10MWT-SSWS and 10MWT-MWS progressions with both SPRS and mSPRS progressions. Open-label studies employing BoNT-A in conjunction with rehabilitation measures (de Niet et al., 2015; Paparella et al., 2020) demonstrated a positive response in both 10MWT-SSWS and in the ClinROs SPRS and Modified Ashworth Scale (MAS), indicating a correlation between improvements in disease severity measured by the SPRS and in the degree of spasticity according to the MAS and improvements in the performance of the 10MWT-SSWS. The lack of response in 10MWT-MWS in a study that showed improvement in MAS (de Niet et al., 2015) and in another study that showed improvement in SPRS (Béreau et al., 2015) did not point to the same type of association when the test is performed at maximum speed. Another explanation for the dissociation of treatment responsiveness between ClinROs and 10MWT-MWS would be that the PerFO, being an objective measure, would be less susceptible to observer measurement biases inherent to open-label studies than SPRS and MAS.

Locomotor rehabilitation index (LRI) takes into account the principle of dynamical similarities and the theory of mechanisms minimizing the energy expenditure in pathological walking, being defined as the percentage ratio between self-selected speed on 10MWT and optimum walking speed (OWS), algebraically LRI = 100 × SSWS/OWS, in which OWS is the square root of the product of gravitational acceleration (9.81 m/s^2^), Froude number (0.25), and lower limb length in meters (Peyré-Tartaruga and Monteiro, 2016). The LRI results were similar to the 10MWT-SSWS. We did not find other studies that evaluated LRI in HSPs so that we could perform comparisons.

In summary, both the 10MWT and the LRI exhibit sensitivity to change among patients with HSPs, with indications of greater sensitivity when the 10MWT is performed at SSWS in analyses that encompass the study’s follow-up time. Additional studies with larger sample sizes and in different COUs (initial disease and specific subtypes of HSP) that use new strategies to anchor the progression or response to treatments with PROs or a qualitative view of patients are needed to define the role of 10MWT and the LRI as endpoints recognized by regulatory agencies for future clinical trials.

#### Timed-up and go

After 3 and 4.5 years of follow-up, significant progressions were found for TUG-SSWS and TUG-MWS in the overall HSP group, while when modeled by disease duration significant annual progression was only found for TUG-MWS. The TUG was initially validated to be applied at SSWS (Podsiadlo and Richardson, 1991), and the other studies that evaluated this PerFO in HSPs performed the test in this condition. The aforementioned study by Regensburger et al. showed statistically significant progression in TUG-SSWS after 14 months of follow-up in complex HSP (Regensburger et al., 2022). Open-label trials that evaluated the effectiveness of BoNT-A associated with rehabilitation measures in HSPs also used TUG-SSWS as an endpoint, with negative results in the Dutch study (de Niet et al., 2015), whereas in the Italian study, there was a reduction of 2.4 s in the median of TUG-SSWS after 3 months of treatment (Paparella et al., 2020). We did not find significant correlations between TUG progression in the two conditions and ClinRO progression, which may indicate a difference in sensitivity to change between instruments. Similarly, in the study by de Niet et al. an improvement was observed in the level of spasticity as measured by MAS, with no effect on TUG-SSWS. On the contrary, improvement in TUG-SSWS performance was accompanied by a reduction in the severity of the disease measured by the SPRS in the study by Paparella et al. Considering the combined data, TUG is sensitive to change in HSPs, with our results pointing to greater sensitivity to change both for the classic form of application of the TUG-SSWS and for the application at MWS. In addition, TUG-MWS was the COA that would require the smallest sample size if it was chosen as the primary endpoint for a clinical trial (Supplementary Table S3) and was the only COA capable of differentiating subjects with a worse compared to a stable/better impression of change in CGI, with its MCID being estimated at 0.15 s, which is 2.5 times greater than the mean annual progression of the test of 0.06 s. The clinical relevance of TUG is well-established through studies involving various medical conditions and elderly populations. It serves as a proxy for the risk of falls, with performances greater than 12 s associated with gait disturbance and above 13.5 s associated with an increased risk of falls (Trueblood et al., 2001; Bischoff et al., 2003; Barry et al., 2014). The median time to perform the TUG in both conditions within our sample exceeded the thresholds established in the literature for the risk of falling (Table 2). It is plausible that additional impairments in this test’s performance are of clinical relevance, which is corroborated by the analysis anchored to CGI-. Thus, TUG can serve as a valuable tool for gauging the progression of HSPs in patients with moderate disease stages. However, additional studies with larger sample sizes and in different COUs, as well as studies that use new strategies to anchor the progression or response to treatments with a PRO or qualitative view of patients, are needed to complement the understanding of the relevance to the patient of changes in this PerFO.

#### 6-min walking test

The 6-min walk test (6MWT) is a well-established PerFO in a variety of diseases, having originally been developed as an integrated global assessment of cardiac, respiratory, circulatory, and muscular capacity (McDonald et al., 2013). 6MWT has been used to evaluate functional capacity and endurance and has been the basis for the regulatory registration of different drugs for neuromuscular diseases (van der Ploeg et al., 2010; Haas et al., 2015; McDonald et al., 2017). We found differences after 4.5 years of follow-up in the 6MWT, and in disease duration modeling, its annual progression was estimated to be 7.71 m in the overall HSP group, 9.12 m in the SPG4 subgroup, and 9.74 m in the subgroup with adult-onset disease. We did not find studies that employed 6MWT in the context of HSPs; however, the study by Paparella et al. evaluated the effect of treatment with BoNT-A associated with intensive physical therapy in the 2-min walk test (2MWT), which can be used to predict the 6MWT (Witherspoon et al., 2019). The authors demonstrated a median increase of 21 m after 1 month of treatment when compared to baseline (Paparella et al., 2020). We found no significant correlation between 6MWT and ClinROs progressions, and 6MWT progression in the present study was not able to differentiate patients with a worse compared to a stable/better impression of change in CGI, and it was also not possible to estimate its MCID. Studies in different neuromuscular conditions indicate that the MCID of 6MWT is a difference greater than 30 m (McDonald et al., 2013, 2017). Considering the annual progression found in 6MWT, it would take approximately 4 years to capture clinically relevant differences according to these studies thresholds, which would be in line with the sample calculation estimates (Supplementary Table S3) that indicated prohibitive sample sizes for ultra-rare diseases if this outcome was chosen as the primary endpoint for studies with disease-modifying drugs. In summary, the 6MWT exhibits sensitivity to changes in HSPs; however, this sensitivity seems to be lower compared to other PerFOs. Additionally, the 6MWT is a more time-consuming and demanding test that requires a larger physical space for accurate execution. Despite this, additional studies are needed with larger sample sizes and in different COUs that use new strategies to anchor the progression or response to treatments with a PRO or qualitative view of patients to define the role of the 6MWT or 2MWT as an endpoint recognized by regulatory agencies for future clinical trials.

### Study limitations

The major study limitation is the small sample size, which is explained by the disease rarity and because this was a single-center initiative. Some patients were lost to follow-up, mainly due to social issues in our region, in which many patients live in the countryside and do not have easy access to the research center, and because this study was conducted during the COVID-19 pandemic. Even so, this is one of the longest studies with the highest number of evaluations and a prospective design that evaluated the progression of COAs in HSPs. It should be borne in mind that the results of this study must be interpreted in the context of use (COU) of a sample composed of adults mostly with pure forms of HSP in moderate stages, in which more than half of the subjects used walking aid assistance.

## Conclusion

Both performance and clinician-reported outcomes can capture the long-term progression of HSP, with 10MWT and TUG, particularly TUG-MWS, being the COAs with greater sensitivity to change. Future multinational cohorts and clinical trials in different contexts of use will be fundamental for defining which COAs should be recognized by regulatory agencies for use in forthcoming clinical trials testing disease-modifying and symptomatic treatments for HSPs.

## Data availability statement

The raw data supporting the conclusions of this article will be made available by the authors, without undue reservation.

## Ethics statement

The studies involving humans were approved by the Ethics in Research Committee of HCPA (GPPG-HCPA-2017-0341). The studies were conducted in accordance with the local legislation and institutional requirements. The participants provided their written informed consent to participate in this study.

## Author contributions

DC: acquisition of data, analysis, and interpretation of data, drafting the article and final approval of the version to be submitted. GD and VM: acquisition of data and final approval of the version to be submitted. VL: statistical analysis. RS: revising the article critically for important intellectual content and final approval of the version to be submitted LP-T: conception and design of the study, interpretation of data, revising the article critically for important intellectual content and final approval of the version to be submitted. JS: conception and design of the study, analysis and interpretation of data, drafting the article and final approval of the version to be submitted. All authors contributed to the article and approved the submitted version.

## Supplementary material

SUPPLEMENTARY Figure 1 Progression of ClinROs and PerFOs according to the study follow-up time in the SPG4 subgroup. SPRS: Spastic Paraplegia Rating Scale; mSPRS: Motor Spastic Paraplegia Rating Scale; 10MWT- SSWS: 10-metres walking test at self-selected speed; 10MWT-MWS (s): 10-metres walking test at maximal speeds; TUG-SSWS: Timed- Up and Go at self-selected walking speed; TUG-MWS: Timed- Up and Go test at maximal walking speed; LRI: Locomotor Rehabilitation Index (%); 6MWT: 6-minute walking test. **p* < 0.05.
