# Supplementary figures and images for: Long-term progression of clinician-reported and gait performance outcomes in hereditary spastic paraplegias
Source: Front Neurosci. 2023 Sep 22;17:1226479. doi: 10.3389/fnins.2023.1226479 (PMC10556702; doi:10.3389/fnins.2023.1226479)

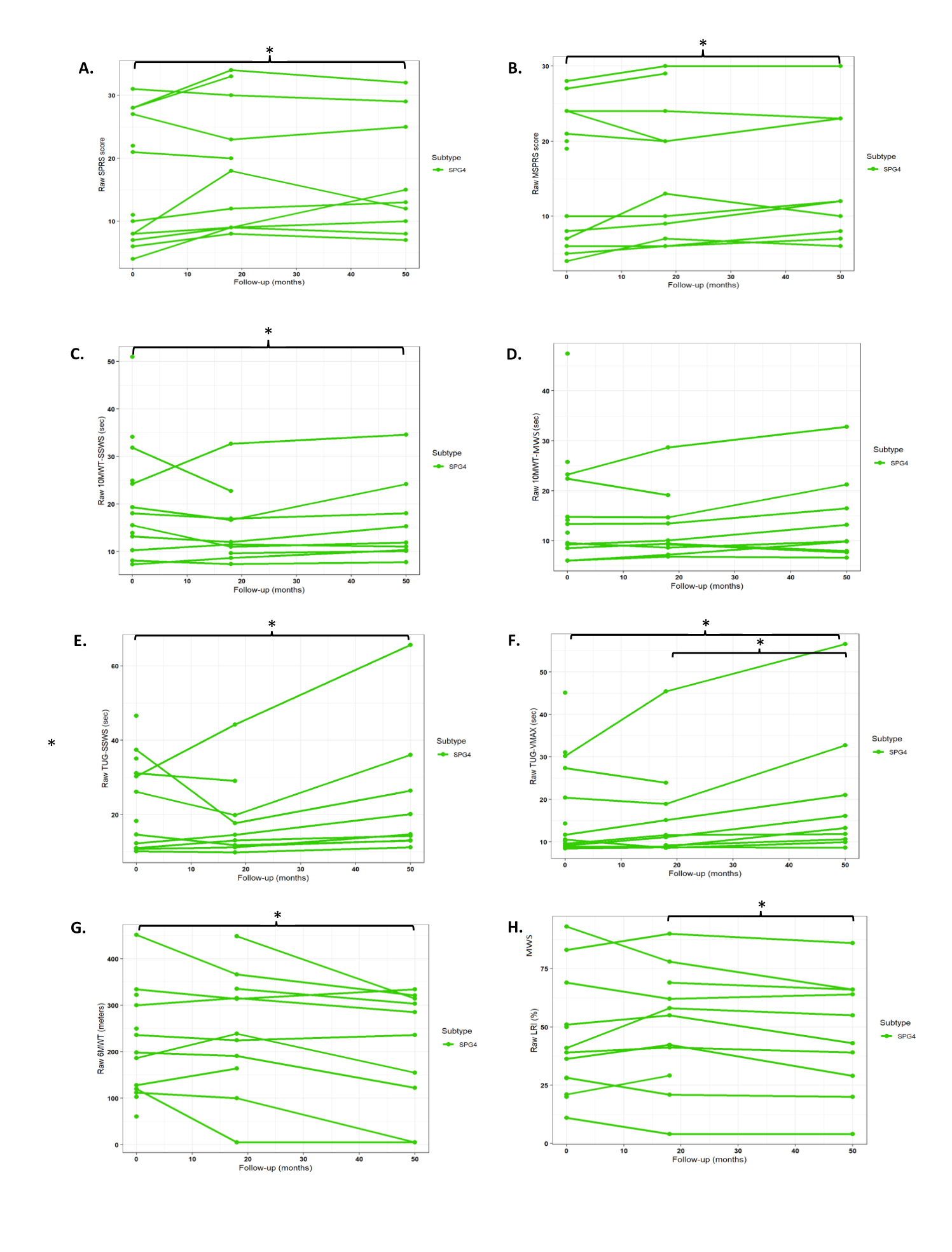

Supplement: SUPPLEMENTARY Figure 1 — Progression of ClinROs and PerFOs according to the study follow-up time in the SPG4 subgroup. SPRS: Spastic Paraplegia Rating Scale; mSPRS: Motor Spastic Paraplegia Rating Scale; 10MWT- SSWS: 10-metres walking test at self-selected speed; 10MWT-MWS (s): 10-metres walking test at maximal speeds; TUG-SSWS: Timed- Up and Go at self-selected walking speed; TUG-MWS: Timed- Up and Go test at maximal walking speed; LRI: Locomotor Rehabilitation Index (%); 6MWT: 6-minute walking test. *p < 0.05. [file Image_1.TIF]
