# Supplementary material for: Long-term progression of clinician-reported and gait performance outcomes in hereditary spastic paraplegias
Source: Front Neurosci. 2023 Sep 22;17:1226479. doi: 10.3389/fnins.2023.1226479 (PMC10556702; doi:10.3389/fnins.2023.1226479)
Supplement: Supplementary file 2 [file Table_1.DOCX]

**SUPP TABLE 1.** Progression of clinical outcome assessments modeled by disease duration.

| **HSP group** | | |
| --- | --- | --- |
| **COA** | **Estimated annual progression** | **p-value** |
| SPRS | 0.43 (CI 95%: 0.096 to 0.774) | 0.019 |
| mSPRS | 0.42 (CI 95%: 0.148 to 0.704) | 0.006 |
| 10MWT-SSWS - s | 0.01 (CI 95%: -0.005 to 0.029) | ns |
| 10MWT-MWS - s | 0.03 (CI 95%: 0.007 to 0.045) | 0.005 |
| TUG SSWS - s | 0.02 (CI 95%: 0.008 to 0.057) | 0.047 |
| TUG-MWS - s | 0.06 (CI 95%: 0.026 to 0.085) | <0.001 |
| 6MWT - m | -7.71 (CI 95%: -12.20 to -3.39) | <0.001 |
| LRI (%) | -0.95 (CI 95%: -1.991 to 0.146) | ns |
| **SPG4 subgroup** | | |
| SPRS | 0.5 (CI 95%: 0.076 to 0.925) | 0.043 |
| mSPRS | 0.45 (CI 95%: 0.142 to 0.791) | 0.017 |
| 10MWT-SSWS - s | 0.02 (CI 95%: -0.001 to 0.042) | ns |
| 10MWT-MWS - s | 0.04 (CI 95%: 0.014 to 0.056) | 0.002 |
| TUG SSWS - s | 0.04 (CI 95%: 0.018 to 0.066) | 0.009 |
| TUG-MWS - s | 0.05 (CI 95%: 0.021 to 0.083) | 0.004 |
| 6MWT - m | -9.12 (CI 95%: -15.21 to -4.856) | 0.006 |
| LRI (%) | -1.26 (CI 95%: -2.664 to 0.098) | ns |
| **Adult-onset HSP** | | |
| SPRS | 0.65 (CI 95%: 0.322 to 0.972) | < 0.001 |
| mSPRS | 0.63 (CI 95%: 0.389 to 0.876) | < 0.001 |
| 10MWT-SSWS - s | 0.03 (CI 95%: 0.014 to 0.054) | 0.002 |
| 10MWT-MWS - s | 0.04 (CI 95%: 0.025 to 0.063) | < 0.001 |
| TUG SSWS - s | 0.05 (CI 95%: 0.023 to 0.067) | < 0.001 |
| TUG-MWS - s | 0.06 (CI 95%: 0.037 to 0.090) | < 0.001 |
| 6MWT - m | -9.74 (CI 95%: -14.24 to -5.011) | < 0.001 |
| LRI (%) | -1.54 (CI 95%: -2.469 to -0.525) | 0.01 |

**Note:** Variables that did not have normal distribution were Log transformed, except for 10MWT-MWS which was box-cox transformed, for the analyses and back transformed to raw values to be presented in the table.**(s):** Seconds; **(m):** meters; **(ns)**: not statistically significant. **CI:** Confidence interval; **COA:** clinical outcome assessments; **SPRS:** Spastic Paraplegia Rating Scale; **mSPRS:** Motor Spastic Paraplegia Rating Scale; **10MWT-SSWS:** 10-metres walking test at self-selected speed; **10MWT-MWS (s):** 10-metres walking test at maximal speeds; **TUG-SSWS**: Timed- Up and Go at self-selected walking speed; **TUG-MWS**: Timed- Up and Go test at maximal walking speed; **6MWT:** 6-minute walking test; **(%):** Percentage; **LRI:** Locomotor Rehabilitation Index.
