# Supplementary material for: Long-term progression of clinician-reported and gait performance outcomes in hereditary spastic paraplegias
Source: Front Neurosci. 2023 Sep 22;17:1226479. doi: 10.3389/fnins.2023.1226479 (PMC10556702; doi:10.3389/fnins.2023.1226479)
Supplement: Supplementary file 3 [file Table_2.DOCX]

**Supplemental Table 2.** The area under the curve for detecting minimal clinically important differences

| **COA** | **AUC (95% CI)** | **p-value** |  |
| --- | --- | --- | --- |
|  |  |  |  |
| Delta_SPRS | 0.457 (95% CI: 0.255 to 0.679) | 0.770 |  |
| Delta_mSPRS | 0.486 (95% CI: 0.359 to 0.798) | 0.486 |  |
| Delta_6MWT | 0.456 (95% CI: 0.215 to 0.696) | 0.701 |  |
| Delta_LRI | 0.550 (95% CI: 0.312 to 0.788) | 0.666 |  |
| Delta_10MWT-SSWS | 0.586 (95% CI: 0.376 to 0.796) | 0.445 |  |
| Delta_10MWT-MWS | 0.576 (95% CI: 0.365 to 0.786) | 0.500 |  |
| Delta_TUG-SSWS | 0.687 (95% CI: 0.490 to 0.884) | 0.096 |  |
| Delta_TUG-MWS | 0.732 (95% CI: 0.547 to 0.918) | 0.039* |  |

**Note:** a. Under the nonparametric assumption, b. Null hypothesis: true area = 0.5

Data are shown as mean and confidence intervals. **COA:** clinical outcome assessments; **TFTs**: timed functional tests of gait; **(s):** Seconds; **(m):** meters; **SPRS:** Spastic Paraplegia Rating Scale; **mSPRS:** Motor Spastic Paraplegia Rating Scale; **10MWT-SSWS:** 10-metres walking test at self-selected speed; **10MWT-MWS (s):** 10-metres walking test at maximal speeds; **TUG-SSWS**: Timed- Up and Go at self-selected walking speed; **TUG-MWS**: Timed- Up and Go test at maximal walking speed; **6MWT:** 6-minute walking test; **(%):** Percentage; **LRI:** Locomotor Rehabilitation Index.*p<0.05
