# Supplementary material for: Long-term progression of clinician-reported and gait performance outcomes in hereditary spastic paraplegias
Source: Front Neurosci. 2023 Sep 22;17:1226479. doi: 10.3389/fnins.2023.1226479 (PMC10556702; doi:10.3389/fnins.2023.1226479)
Supplement: Supplementary file 4 [file Table_3.DOCX]

**Supplemental Table 3.** Sample size estimation for future clinical trials using the disease duration model.

| **COA** | **RELATIVE EFFECT** | **n.Per.Arm** | **COA** | **RELATIVE EFFECT** | **n.Per.Arm** |
| --- | --- | --- | --- | --- | --- |
| **SPRS** | 0.25 | 13449 | **logTUG-SSWS** | 0.25 | 7246 |
|  | 0.5 | 3362 |  | 0.5 | 1811 |
|  | 0.75 | 1494 |  | 0.75 | 805 |
|  | 1 | 840 |  | 1 | 452 |
|  | 1.25 | 537 |  | 1.25 | 289 |
|  | 1.5 | 373 |  | 1.5 | 201 |
|  | 1.75 | 274 |  | 1.75 | 147 |
|  | 2 | 210 |  | 2 | 113 |
| **mSPRS** | 0.25 | 7207 | **logTUG-MWS** | 0.25 | 2317 |
|  | 0.5 | 1801 |  | 0.5 | 579 |
|  | 0.75 | 800 |  | 0.75 | 257 |
|  | 1 | 450 |  | 1 | 144 |
|  | 1.25 | 288 |  | 1.25 | 92 |
|  | 1.5 | 200 |  | 1.5 | 64 |
|  | 1.75 | 147 |  | 1.75 | 47 |
|  | 2 | 112 |  | 2 | 36 |
| **log10MWT-SSWS** | 0.25 | 27319 | **6MWT** | 0.25 | 12786 |
|  | 0.5 | 6829 |  | 0.5 | 3196 |
|  | 0.75 | 3035 |  | 0.75 | 1420 |
|  | 1 | 1707 |  | 1 | 799 |
|  | 1.25 | 1092 |  | 1.25 | 511 |
|  | 1.5 | 758 |  | 1.5 | 355 |
|  | 1.75 | 557 |  | 1.75 | 260 |
|  | 2 | 426 |  | 2 | 199 |
| **bc10MWT-MWS** | 0.25 | 11381 | **LRI** | 0.25 | 19681 |
|  | 0.5 | 2845 |  | 0.5 | 4920 |
|  | 0.75 | 1264 |  | 0.75 | 2186 |
|  | 1 | 711 |  | 1 | 1230 |
|  | 1.25 | 455 |  | 1.25 | 787 |
|  | 1.5 | 316 |  | 1.5 | 546 |
|  | 1.75 | 232 |  | 1.75 | 401 |
|  | 2 | 177 |  | 2 | 307 |

**Note: (s):** Seconds; **(m):** meters; **COA**: clinical outcome assessments; **SPRS:** Spastic Paraplegia Rating Scale; **mSPRS:** Motor Spastic Paraplegia Rating Scale; **10MWT-SSWS:** 10-meter walking test at self-selected speed; **10MWT-MWS (s):** 10-meter walking test at maximal speeds; **TUG-SSWS**: Timed-Up and Go at self-selected walking speed; **TUG-MWS**: Timed-Up and Go test at maximal walking speed; **6MWT:** 6-minute walking test; **(%):** Percentage; **LRI:** Locomotor Rehabilitation Index; **Log:** Logarithm; **bc:** box-cox
